# Multidrug resistant *Kluyvera ascorbata *septicemia in an adult patient: a case report

**DOI:** 10.1186/1752-1947-4-197

**Published:** 2010-06-29

**Authors:** Shannon Moonah, Kavita Deonarine, Clyde Freeman

**Affiliations:** 1 Department of Medicine, Howard University Hospital, 2041 Georgia Avenue NW, Washington DC, 20060, USA

## Abstract

**Introduction:**

*Kluyvera ascorbata *has become increasingly significant due to its potential to cause a wide range of infections, as well as its ability to transfer gene encoding for CTX-M- type extended spectrum B-lactamases (ESBLs) to other Enterobacteriaceae.

**Case presentation:**

We report the case of a 64-year-old African-American male diagnosed with severe sepsis due to a multidrug resistant *Kluyvera ascorbata*, which was isolated from his blood. He was treated with meropenem and had a favorable outcome.

**Conclusion:**

To the best of our knowledge, this is the first case report of a multidrug resistant *Kluyvera ascorbata *isolated from the blood in an adult patient with sepsis.

## Introduction

*Kluyvera ascorbata *is a gram negative microorganism belonging to the family Enterobacteriaceae. Although it causes infections infrequently, it is responsible for causing a wide range of infections including severe sepsis [1,2]. It is believed to be the source of genes encoding CTX-M-type extended spectrum B-lactamases (ESBLs) and it has the ability to transfer these genes to other Enterobacteriacae [3]. Only three cases of *K. ascorbata *isolated from the blood of adult patients have been reported [4-6]. We report what we believe to be the first case of a multidrug resistant *K. ascorbata *isolated from the blood of an adult patient with sepsis.

## Case presentation

A 64-year-old African-American man with a past medical history of hypertension, type 2 diabetes mellitus, bilateral above knee amputation, prostate cancer post radical prostatectomy in 1999, quadraparesis secondary to cervical spine fracture of C4, neurogenic bladder with an indwelling suprapubic catheter and recurrent urinary tract infections was transferred from a nursing home to Howard University Hospital in June 2009 because of lethargy, fever and low blood pressure (BP). There was no history of cough, chest pain, vomiting, diarrhea or headache.

His admitting temperature was 101.4°F and blood pressure 61/34 mmHg, which responded to intravenous fluid boluses. His initial white blood count (WBC) was 14.4×10^9^/L. His chest radiograph showed mild left lung base ateclectasis, but the rest of the lung fields were clear. Urinalysis showed large amounts of red cells, white cells and numerous bacteria. He was admitted to the medical intensive care unit (MICU) and started empirically on vancomycin and levofloxacin.

Over the following five days his condition improved, with normalization of his mental status, temperature, BP and WBC. His initial blood culture bottle grew gram-positive cocci, identified as Coagulase-negative staphylococci, thought to be a contaminant. Both urine cultures were sterile.

He was transferred to the medical floor for further care. 24 hours later he developed a low grade temperature of 95.9°F, his BP decreased to a systolic of 75 mmHg and WBC increased to 13×10^9^/L. There was no change in his mental status. He was given boluses of intravenous fluids. Meropenem was immediately added to his antibiotic regimen. A gram stain of his repeat blood culture revealed gram negative rods which were later identified as *K. ascorbata*. The isolate was susceptible to amikacin, tobramycin and imipenem, but resistant to ampicillin, piperacillin, cefazolin, cefuroxime, cefotaxime, ceftriaxone, ceftazidime, aztreonam, ciprofloxacin and levofloxacin. Species identification and antimicrobial susceptibility testing was performed using Microscan panels (Dade Behring). He was placed on contact isolation and levofloxacin was discontinued. Over the next five days he maintained a normal temperature, BP and WBC. Repeat blood cultures and a urine culture were negative for growth. He was discharged back to the nursing home after 13 days of hospitalization for continued care.

## Discussion

*Kluyvera *spp was first described in 1936 by Kluyver and van Neil [7], but it was not until 1981 that it was defined completely using molecular characterization [8]. Four species are described: *K. cryocrescens, K. ascorbata, K. georgiana*, and *K. cochleae. K. ascobata *causes a wide range of infectious diseases in different age groups and of varying severity [1,2,9].

Only three cases isolating *K. ascobata *from the blood of adult patients with sepsis have been reported. In all three cases the organism was susceptible to third generation cephalosporins (Table 1). To the best of our knowledge, this is the first case report describing an isolate of multidrug resistant *K. ascorbata *from the blood of an adult patient with sepsis. The isolate was resistant to third generation cephalosporins and fluoroquinolones. In addition to its ability to cause severe sepsis, we also report its multidrug resistant potential. This must be considered when choosing appropriate antimicrobial therapy. We believe that the prompt administration of a carbapenem resulted in a favorable outcome for the patient.

**Table 1:** Summary of the four reported *Kluyvera ascorbata *cases isolated from the blood of adult patients

Ref.	Age/sex	Past medical history	Antimicrobial susceptibility		Treatment	Outcome
			Susceptible	Resistant		
[4]	72/M	Liver cirrhosis, Hepatocellular carcinoma, Hepatitis C	Amoxicilin/Clavulanate 3^rd ^generation cephalosporins Aminoglycosides Ciprofloxacin Imipenem Aztreonam	Ampicillin Ticarcillin Cephalothin Cefuroxime	Cefotaxime	Recovered
[5]	23/M	Liver cirrhosis, Hepatitis B	Amoxicilin/ Clavulanate Piperacillin Ceftriaxone Gentamicin Ciprofloxacin	Ampicillin Cefazolin Ticarcillin	Ceftriaxone	Recovered
[6]	57/F	Colon adenocarcioma, Chemotherapy, Neutropenia	Aminoglycosides 3^rd ^generation cephalosporins Flouroquinolones Ureidopenicillins	Ampicillin Amoxicilin/ Clavulanate 2^nd ^generation cephalosporins Cotrimazole	Ceftazidime Amikacin	Expired
Present report	64/M	Neurogenic bladder with an indwelling suprabupic catheter, Recurrent urinary tract infections	Amikacin Tobramycin Imipenem	Ampicillin Aztreonam Ceftazidime Cefotaxime Cetftriaxone Cefuroxime Cefazolin Ciprofloxacin Levofloxacin Piperacillin	Meropenem	Recovered

ESBLs are enzymes produced by certain types of bacteria such as *E. coli*. They mediate resistance to extended-spectrum cephalosporins (e.g. ceftriaxone) but do not affect carbapenems (e.g. meropenem). Molecular and genetic evidence indicates that CTX-M-type ESBLs found in *E. coli *and other Enterobacteriaceace evolved from chromosomal genes from *K. ascobata*. In the past decade CTX-M enzymes have become the most prevalent ESBLs and CTX-M producing *E. coli *is becoming a major public health problem. This rise will result in the narrowing of effective options to treat infections caused by these organisms. There will likely be increased usage of carbapenems, thus generating further selective pressure for carbapenemases and carbepenem resistance in the future [3,10-12].

## Conclusion

*K. ascorbata *is an infrequent cause of infection, but can result in severe sepsis. Clinicians should be aware of its infectious and multidrug resistant potential as early and appropriate treatment can result in recovery.

## Consent

Written informed consent was obtained from the patient for the publication of the case report and any accompanying images. A copy of the written consent is available for review by the Editor-in-Chief of this journal.

## Competing interests

The authors declare that they have no competing interests.

## Authors' contributions

SM, KD, and CF were involved in the direct care of the patient, and contributed to the literature search, data collection, data analysis, and manuscript preparation. All authors have read and approve of the submitted manuscript.
